# Segregation of a Latent High Adiposity Phenotype in Families with a History of Type 2 Diabetes Mellitus Implicates Rare Obesity-Susceptibility Genetic Variants with Large Effects in Diabetes-Related Obesity

**DOI:** 10.1371/journal.pone.0070435

**Published:** 2013-08-07

**Authors:** Arthur B. Jenkins, Marijka Batterham, Dorit Samocha-Bonet, Katherine Tonks, Jerry R. Greenfield, Lesley V. Campbell

**Affiliations:** 1 School of Health Sciences, University of Wollongong, Wollongong, NSW, Australia; 2 Diabetes and Obesity Research Program, Garvan Institute of Medical Research, Sydney, NSW, Australia; 3 Centre for Statistical and Survey Methodology, University of Wollongong, Wollongong, NSW, Australia; 4 Diabetes Centre and Department of Endocrinology, St Vincent's Hospital, Sydney, NSW, Australia; University of Milan, Italy

## Abstract

**Background:**

We recently reported significantly greater weight gain in non-diabetic healthy subjects with a 1^st^ degree family history (FH+) of type 2 diabetes mellitus (T2DM) than in a matched control group without such history (FH−) during voluntary overfeeding, implying co-inheritance of susceptibilities to T2DM and obesity. We have estimated the extent and mode of inheritance of susceptibility to increased adiposity in FH+.

**Methods:**

Normoglycaemic participants were categorised either FH+ (≥1 1^st^ degree relative with T2DM, 50F/30M, age 45±14 (SD) yr) or FH− (71F/51M, age 43±14 yr). Log-transformed anthropometric measurements (height, hip and waist circumferences) and lean, bone and fat mass (Dual Energy X-ray Absorptiometry) data were analysed by rotated Factor Analysis. The age- and gender-adjusted distributions of indices of adiposity in FH+ were assessed by fits to a bimodal model and by relative risk ratios (RR, FH+/FH−) and interpreted in a purely genetic model of FH effects.

**Results:**

The two orthogonal factors extracted, interpretable as *Frame* and *Adiposity* accounted for 80% of the variance in the input data. FH+ was associated with significantly higher *Adiposity* scores (p<0.01) without affecting *Frame* scores. *Adiposity* scores in FH+ conformed to a bimodal normal distribution, consistent with dominant expression of major susceptibility genes with 59% (95% CI 40%, 74%) of individuals under the higher mode. Calculated risk allele frequencies were 0.09 (0.02, 0.23) in FH−, 0.36 (0.22, 0.48) in FH+ and 0.62 (0.36, 0.88) in unobserved T2DM-affected family members.

**Conclusions:**

The segregation of *Adiposity* in T2DM-affected families is consistent with dominant expression of rare risk variants with major effects, which are expressed in over half of FH+ and which can account for most T2DM-associated obesity in our population. The calculated risk allele frequency in FH− suggests that rare genetic variants could also account for a substantial fraction of the prevalent obesity in this society.

## Introduction

Type 2 diabetes mellitus (T2DM) is a growing worldwide public and individual health burden, linked to a similar increase in overweight and obesity [1], which is present in 80–90% of T2DM [2]. Increased adiposity and T2DM are both under strong genetic influences but the precise links, genetic and otherwise, between the two conditions are not clarified [3]. The known common single nucleotide variants (SNVs) account for small fractions of the total genetic susceptibilities to increased adiposity (<5%) and T2DM (<10%) [4]. The much-sought cause(s) of the missing heritability in genome-wide association studies (GWAS) is unclear but possible contributors include small effect sizes, copy number variants (CNVs), rare variants, gene-gene and gene-environment interactions and poor definitions of phenotypes in GWAS [5].

Reports of higher BMI in individuals with a family history of T2DM [6], [7] imply that susceptibilities to increased adiposity and to T2DM are co-transmitted in families. Recently, we showed that healthy non-diabetic subjects with a family history of T2DM gained significantly more weight than a matched control group without a family history during a month-long voluntary overfeeding protocol [8]. We then aimed to test, in a similar healthy but larger group, if family history of T2DM has an effect on accurately measured body composition consistent with co-transmission of the two traits.

The known genetic components of both T2DM and increased adiposity are highly heterogeneous [9] and are often characterised as polygenic in nature, where individual genetic susceptibility to the disorder is a result of the combined actions of multiple susceptibility variants at different loci [10]. Under those conditions transmission of susceptibility will not follow simple Mendelian patterns. Association studies are not powered to detect the predicted interactions between multiple loci. Recent evidence indicates that most of the variation in the human genome is rare and therefore below detection threshold for association studies [11]–[14]. This raises the strong possibility that many complex disorders have a heterogenetic basis in which individual genetic susceptibility is strongly determined by a single rare variant which would often differ between unrelated individuals [15]. While both heterogenetic and polygenetic mechanisms could explain the minor fraction of overall genetic susceptibility currently detected by genomic studies [4], they differ in prediction regarding segregation analyses. Importantly, in contrast to polygenetic causal variants, a single locus heterogenetic model predicts Mendelian segregation of susceptibility within families [5].

Genetically complex disorders like increased adiposity and T2DM may also be phenotypically complex but usually the choice of phenotypic markers for expensive large scale genetic studies is based on logistic considerations alone. Simplistic clinical management phenotypes do not adequately represent the genetic underpinnings of these disorders and hence contribute to the apparent complexity of the current picture [5], [16]. The concept of increased adiposity appears intuitively simple, but in humans is not easy to define or measure accurately. Most genetic studies of adiposity use the surrogate BMI, despite its well-recognised limitations as a measurement of true adiposity [16]–[18], because potentially more informative measures have been impractical for large samples. Similar limitations also apply to common anthropometric indices involving waist and hip circumferences. More direct measures of body fat content have been used in some studies (e.g. Pecioska et al. [19]), but it is not clear *a priori* how to express these measures as a biologically meaningful index of increased adiposity. The common clinical usage of percent body fat cut-offs is arbitrary [20] and analysing continuous measures like percent body fat or fat mass indices implies assumptions about how adiposity affects or is affected by disease processes. The use of either BMI or percent body fat as covariates can lead to erroneous conclusions in a genetic context [21].

A less arbitrary approach is the use of factor analysis, a statistical technique that extracts a small number of latent (unmeasured) factors that account for the correlations between multiple related variables. Our data, consisting of direct measures of fat, lean and bone masses as well as anthropometric measures (height, waist and hip circumferences) are well suited to factor analysis. We have extracted from our data a factor interpretable as *Adiposity* and analysed its relationship to family history of T2DM in a model which allows discrimination between polygenetic and heterogenetic modes of inheritance.

## Methods

### Ethics Statement

The studies were approved by the Human Research and Ethics Committee of St Vincent's Hospital, Sydney. All participants provided written informed consent.

### Recruitment, Selection and Matching

Participants (n = 202) were recruited, by advertisements in newspapers and around the St. Vincent's Hospital Sydney campus, into studies conducted over the period 1994–2010 at the Garvan Institute [8], [22]–[25]. Participants with one or more first degree relatives diagnosed with T2DM were categorised FH+. Within studies, FH+ and FH− participants were matched for gender, age and BMI. In the combined sample, gender- and age-matching were preserved with borderline matching of BMI (Table 1). Subjects were excluded if weight had changed substantially in the preceding 3 to 6 months, if they exercised more than 60 min per week, if they were taking medications known to affect insulin sensitivity or if they had a personal history of T2DM. Our data set consists of de-identified data from all participants for whom complete birth date, gender, body composition, anthropometry and FH data were available as of 29/10/2010.

**Table 1:** Effects of family history of T2DM (FH+/−) on anthropometric and body composition variables.

	Group^a^		FH effect^b^	
Variable	FH+	FH−	Unadjusted	Adjusted^c^
Gender (F/M)	50/30	71/51	0.54	–
Age (y)	45±14	43±14	0.34	–
Height (m)	1.69±0.10	1.70±0.10	0.72	0.81
Waist (cm)	89±13	85±15	0.06	**0.01**
Hip (cm)	103±9	101±11	**0.05**	0.06
Body weight (kg)	76±15	74±17	0.25	0.12
Lean mass (kg)	47±11	47±11	0.61	0.99
Bone mass (kg)	2.8±0.5	2.8±0.5	0.47	0.11
Fat mass (kg)	27±10	24±12	**0.04**	**0.05**
BMI (kg/m^2^)	26.7±4.5	25.5±5.3	0.06	0.06
Normal/O'weight /Obese^d^ (%)	38/40/23	55/30/15	**0.05**	–
Body fat (%)	35.3±9.6	31.5±10.5	**0.01**	**0.01**
*Frame* (SD units)	−0.07±1.00	0.05±1.01	0.41	0.57
*Adiposity* (SD units)	0.25±0.88	−0.16±1.09	**0.01**	**0.01**

a Mean ± SD except for Gender (N) and BMI category (%).

b p from ANOVA except for Gender and BMI category (Chi square).

c Adjusted for Gender and Age tertile.

d BMI <25 (Normal), 25–29.9 (Overweight), ≥30 (Obese) kg/m^2^.

### Measurements

#### Anthropometry

Weight and height were measured in light clothing with footwear removed. Body mass index (BMI) was calculated as weight divided by height squared (kg/m^2^). With the subject standing, waist circumference at the level of the umbilicus and hip circumference at the level of the greater trochanter were measured to the nearest 0.5 cm using a flexible tape.

#### Body composition

Fat mass, lean mass and bone mass were assessed by Dual Energy X-ray Absorptiometry (DXA; Lunar DPX-Lunar Radiation V1.3y-1.35y, Madison, WI, USA).

### Data Analysis

#### Model

The model of family history that we use is based on the classic ADCE model for twin data, where total trait variance is partitioned into either additive or dominant genetic (A or D) and persistent shared (C) and individual (E) environmental components [26]. E includes all error variance. Gene-environment interactions are clearly important for the expression of increased adiposity but are difficult to quantify and their presence degrades the ability of genetic analyses to isolate both gene and environmental effects. The increase in prevalence of overweight and obesity over the last half century is too large and too rapid to be due to changes in the gene pool and must somehow reflect the effect of deleterious environmental influences [1]. However, current data suggest that the prevalence of obesity is plateauing in some highly developed countries, including Australia [27], [28], consistent with the notion that the full expression of genetic susceptibility is being approached in the worst affected populations. Under such circumstances, gene-environment interactions have permitted increasing expression of underlying genetic susceptibility and the interactions will collapse towards pure gene effects [29], simplifying the analysis of genetic data. We therefore assume that the effects of gene-environment interactions are saturated in our data and that the effects of FH can be represented as the sum of the A or D and C components.

Because of the close association between T2DM and increased adiposity, a family history of T2DM represents the sum of genetic and persistent shared environmental influences on T2DM risk and on risk of increased adiposity. Although the effects of any persistent shared environmental influences have been difficult to quantify, the relevant literature consistently demonstrates that those influences are negligible on traits related to body composition or T2DM: ie studies of Body Fat% [30] and BMI [31]–[33] in twins, BMI [34] and T2DM [35] in adoptees and in appetite-related traits in children [36]. Hence, in the current context, the effects of family history can be interpreted as predominantly genetic. The total trait variance is therefore modelled as A or D + E, and heritability (h^2^) is obtained from:
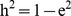
where e^2^ is the proportion of error variance in the models incorporating FH effects.

Under these assumptions, a heterogenetic model of inheritance predicts segregation of traits in families [5] and we therefore analysed and compared the distributions of traits in the FH+ and FH− groups.

#### Statistical methods

All analyses were performed using R 2.12.1 [37].

Effects of Age (as tertiles) and Gender were assessed by two-way ANOVA. No significant interactions between age and gender were detected in any models (p>0.35). BMI was analysed after Log-transformation to correct markedly skewed residuals.

Two orthogonal (uncorrelated) factors were extracted from the log-transformed anthropometric (height, waist and hip circumferences) and body composition (lean, bone and fat masses) data using the factanal function in R with varimax rotation. This procedure produces the most parsimonious orthogonal factors. The input variables were log-transformed to accommodate the geometric relationships between measurements in one linear dimension (height and circumferences) and those in 3 dimensions (masses, which are related to volumes) [21]. Predicted individual scores (SD units) on the factors were calculated using Bartlett's method in factanal.

Effects of a family history of T2DM (FH+, FH−) were assessed initially by three-way ANOVA (Gender + Age tertile + FH). No significant two-way interactions were detected in any models (p>0.13). Further analyses of FH effects were conducted on variables adjusted for the effects of gender and age (coded and presented as residuals from Gender + Age tertile models). Effects of FH were also assessed by comparing distributions of Gender- and Age-adjusted variables between FH+ and FH− groups: variables were binned by deciles of the full sample and relative risks (RR, FH+/FH−) of occurrence in each decile were calculated as p_i_(FH+)/p_i_(FH−) where p_i_  =  n_i_/N, n_i_  =  individuals in the ith decile and N  =  individuals per group; 95% confidence intervals were obtained from the standard error of log(RR) assuming normality.

Gender- and Age-adjusted variables were fitted to bimodal normal distributions using the optim function in R. Data were binned by deciles of the full sample and bin densities were fitted to the model:
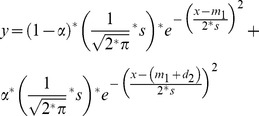
where: y  =  predicted density of the bimodal model at the mid-point of each decile of x, x  =  empirical density at the mid-point of each decile, m_1_  =  mean of the lower mode, d_2_  =  positive difference between the means of the two modes, s  =  standard deviation, α  =  fraction of the total density accounted for by the upper mode.

α was constrained to the interval 0–1, as was d_2_ to ≥0, using the L-BFGS-B method in optim. Heritability (h^2^) was calculated as 1-s^2^.

An estimate of the fraction of FH− individuals in the upper mode was obtained from the relationship
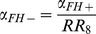
where α _FH−_ and α_FH+_ are respectively the fractions of the FH− and FH+ groups under the upper mode, and RR_8_ is the relative risk ratio (FH+/FH−) in the 8^th^ decile of the full distribution.

Risk variant frequencies in FH− and FH+ groups, were calculated from the phenotypic proportions (α_FH−_ and α_FH+_) under a dominant bi-allelic model of inheritance assuming Hardy-Weinberg equilibrium [38]. In this model, genotype frequencies (AA, Aa, aa) are related to allele frequencies (p, q) through the relationships AA = p^2^, Aa  = 2pq, aa  = q^2^, where a and q represent the high *Adiposity* risk alleles, and α = 2pq +q^2^. It follows that:
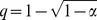



Risk variant and genotype frequencies in the unobserved T2DM-affected family members were calculated assuming that the FH− group represents the pool of spouses in the parental generation i.e.
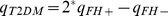



95% confidence intervals for parameter estimates and derived frequencies were obtained by bootstrap re-sampling (N_FH−_ = 122+ N_FH+_ = 80, 1000 draws).

## Results

Participants with (FH+) and without (FH−) a family history of T2DM were well matched for age and gender (Table 1). After adjustment for the age and gender effects illustrated in Table 2, the FH+ group had significantly higher percentage body fat (Body Fat%), fat mass and waist circumference, with borderline higher hip circumference and continuous and categorical BMI (Table 1).

**Table 2:** Anthropometry, body composition, Frame and Adiposity factor scores by gender and age tertile. Mean ± SD.

Gender	Female			Male				
Age Tertile	1	2	3	1	2	3	ANOVA effects (p)^a^	
n	41	44	36	25	26	30	Gender	Age
**Age (y)**	27.5±5.0	43.9±4.8	60.0±5.4	29.5±2.9	42.7±4.6	61.3±5.1	0.30	–
**Height (m)**	1.66±0.07	1.63±0.06	1.61±0.06	1.79±0.06	1.79±0.08	1.76±0.07	**<0.0001**	**0.0007**
**Waist (cm)**	75±10	81±12	88±14	85±10	96±10	100±14	**<0.0001**	**<0.0001**
**Hip (cm)**	98±9	102±13	105±11	99±8	102±6	104±9	0.88	**0.001**
**Body weight (kg)**	66.3±12.4	67.8±14.0	71.6±14.2	79.0±13.2	85.7±13.9	86.3±18.5	**<0.0001**	0.11
**Lean mass (kg)**	39.9±3.8	39.2±4.6	39.8±4.2	58.8±6.7	57.5±7.3	58.4±7.9	**<0.0001**	0.90
**Bone mass (kg)**	2.7±0.3	2.5±0.3	2.4±0.3	3.3±0.5	3.2±0.4	3.1±0.5	**<0.0001**	**0.004**
**Fat mass (kg)**	24.2±10.3	26.3±11.3	30.0±11.8	17.7±8.7	25.8±8.8	26.1±12.6	**0.02**	**0.02**
**BMI (kg/m^2^)** b	24.3±4.3	25.6±5.6	28.1±5.6	24.8±3.4	26.8±41.	28.1±5.8	0.35	**0.001**
**Body fat (%)**	34.9±8.2	37.2±9.8	40.3±8.6	21.3±7.7	29.2±6.4	28.4±8.4	**<0.0001**	**0.006**
***Frame*** ** (SD units)**	−0.64±0.40	−0.76±0.50	−0.75±0.44	1.20±0.51	0.98±0.54	1.04±0.56	**<0.0001**	0.32
***Adiposity*** ** (SD units)**	−0.24±0.89	0.05±1.14	0.45±0.97	−0.71±1.01	0.16±0.74	0.18±1.05	0.14	**0.003**

a No significant Gender*Age interactions (all p>0.35).

b Analysed after log-transformation.

**Figure 1:**
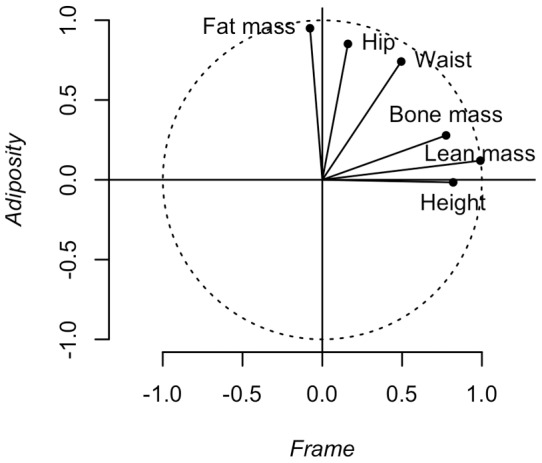
Loading pattern of log-transformed body size and composition variables on the ‘*Frame*’ and ‘*Adiposity*’ factors. The loadings are the correlation coefficients between the input variables and the extracted factors.

The two factors extracted from the anthropometric and body composition data (*Frame* and *Adiposity*) accounted for 80% and 95% of the standardised variance and covariance respectively in the input variables and provide a readily interpretable summary of the data. The loading pattern of the input variables on the two factors (Fig. 1) suggests *Frame* and *Adiposity* as appropriate names for the factors. *Frame* displays the expected large effect of gender, but no effect of age (Table 2). *Adiposity* was not affected by gender in this sample but increased significantly with age (Table 2). After adjustment for age and gender *Frame* was not affected by FH but *Adiposity* was significantly higher in FH+ (Table 1). *Adiposity* correlated strongly and equally well with Body Fat % (r = 0.91) and log-transformed BMI (r = 0.93) (all adjusted for age and gender).

**Figure 2:**
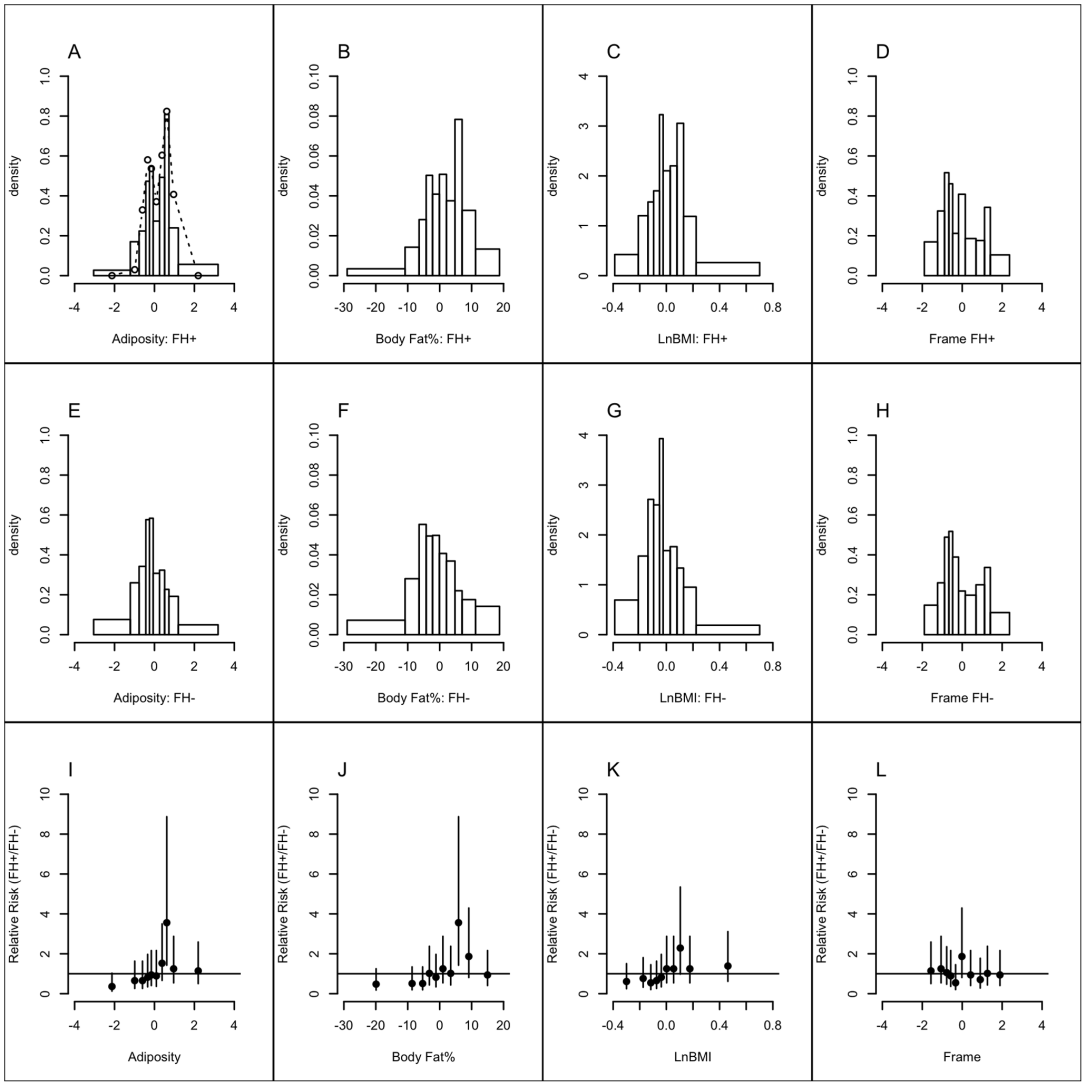
Distributions of gender- and age-adjusted body composition variables. A–D: FH+; E–H: FH−; I–L: relative risks of occurrence (with 95%CI) in each decile of the distributions of body composition variables (FH+/FH−) The dotted line in panel A represents the fit to a bimodal normal distribution.

**Figure 3:**
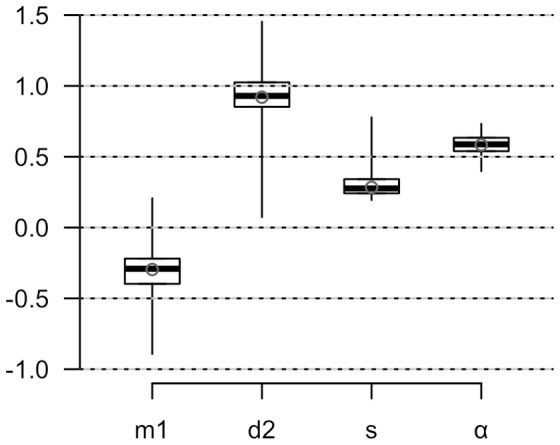
Quantile boxplots representing the median, interquartile range and 95% confidence limits of the parameters of the bimodal distribution model obtained from the *Adiposity* data in FH+ by bootstrap re-sampling. m1  =  mean of the lower mode; d2 =  difference between the means of the 2 modes; s  =  common standard deviation; α  =  the fraction of individuals in the higher distribution. The open circles are the estimates from the original data.

The distributions of the measures of adiposity (*Adiposity*, Body Fat%, log-transformed BMI) and *Frame* in FH+ and FH− groups are compared in Fig 2. Compared to FH−, FH+ distributions for the adiposity measures show a localised excess density around the 8^th^ decile, consistent with bimodality, which was most apparent in *Adiposity*; *Frame* was distributed similarly in the two groups. The relative risks of occupancy of the deciles (Fig 2 I–L) illustrate this pattern, with localised enrichments of FH+ in the 8^th^ decile of *Adiposity* (RR 3.6 95%CI 1.4, 8.9) and Body Fat %, (3.6 1.4, 8.9), a similar tendency in log-transformed BMI (2.3 1.0, 5.4) and no significant enrichment in any decile of *Frame*. Fits to a bimodal distribution identified a 2^nd^ mode only in the *Adiposity* data from FH+ (Fig 2A, Fig 3), with the distance between modes of 0.93 (95%CI 0.07, 1.45) SD units and an estimated 59% (95%CI 40%, 74%) of individuals under the upper mode (α, Fig 3). Heritability of age- and gender-adjusted *Adiposity* calculated from the standard deviation was 91% (31%, 96%); when expressed in terms of unadjusted *Adiposity* by including the contributions of age and gender (R^2^ = 0.12) the estimate was 80% (27%, 84%).

The bimodal distribution of *Adiposity* in FH+ is consistent with dominant expression of susceptibility variants in a bi-allelic system. Table 3 shows the estimated phenotypic proportions (α) and risk allele frequencies (q) in FH−, FH+ and the unobserved T2DM-affected family members, assuming dominance and Hardy-Weinberg equilibrium. The analysis predicts that approximately 85% of the T2DM individuals carry a dominantly-expressed genetic risk of increased adiposity.

**Table 3:** Phenotypic proportions (α) and estimated risk allele frequencies (q) in FH−, FH+ and unobserved T2DM-affected family members under a dominant model of inheritance of Adiposity.

	FH−	FH+	T2DM^a^
α^b^	0.16 (0.04, 0.41)	0.59 (0.40, 0.74)	0.85 (0.60,0.98)
q^c^	0.09 (0.02, 0.23)	0.36 (0.22, 0.48)	0.62 (0.36, 0.88)

Presented as median (95% CI).

a calculated from q _FH−_ and q_FH+_ assuming that the FH− group represents the pool of spouses in the parental generation.

b Fraction of individuals under the higher *Adiposity* mode.

c calculated assuming a dominant bi-allelic model of inheritance in Hardy-Weinberg equilibrium.

## Discussion

### Summary

In our sample approximately half of the individuals with a 1^st^ degree family history of T2DM (FH+) manifest a susceptibility to increased adiposity. This is consistent with the greater weight gain of FH+ drawn from the same population during voluntary over-feeding [8], and with previous reports of association between FH+ and increased BMI [6], [7]. We conclude that this genetic susceptibility to increased adiposity is mediated at least in part by behavioural responses to food-related signals, consistent with the likely effects of the known obesity-related genetic variants [9]. The distribution of *Adiposity* in FH+ is not consistent with the predictions of a polygenic model but conforms to a heterogenetic model of dominant expression of rare susceptibility variants with large effects. The predicted 86% of unobserved T2DM individuals carrying these variants is close to the estimated 80–90% prevalence of overweight/obesity in T2DM [2]. We hypothesise that most or all of the overweight/obesity in T2DM is due to a dominantly expressed genetic susceptibility to increased adiposity. The same mechanism could account for a substantial fraction of endemic obesity.

### Segregation and mode of inheritance of increased Adiposity

Many previous studies have detected evidence of segregation and/or multimodality of adiposity-related traits but the inferred modes of inheritance have varied. Early studies (reviewed in Price et al [39]) found evidence of recessive expression of higher adiposity, while more recent studies have tended to favour dominant or additive models of expression. As argued by Price et al [39], part of this variation may be may be due secular trends in gene-environment interactions which have the effect of simulating recessive inheritance in multi-generational studies. The use of extreme obesity phenotypes would also tend to favour a recessive pattern if the true mode is additive and the heterozygous phenotype is either obscured by definition [40] or is indistinguishable in the data [41], [42]. Finally, very large sample sizes are required to reliably detect dominance effects and some recessive findings are likely to be due to lack of power to detect other modes of inheritance [43]. In summary, published studies are not inconsistent with our interpretation of dominant or additive expression of genetic susceptibility to increased adiposity and some reports provide explicit support for it [39], [44]–[47].

The bimodal distribution of *Adiposity* in FH+ is consistent with segregation in families of obesity susceptibility variants with major effects, in contrast to the predictions of a polygenic model in which variants at many loci have cumulative small effects on the phenotype. Previous studies of the transmission of obesity-related phenotypes have also generally not favoured the polygenic model. The inability of common SNVs to account for more than a small fraction of the heritability of increased adiposity in GWAS implies that the variants responsible for segregation must have escaped detection and are therefore either rare, or are not closely linked to the common SNVs targeted in GWAS, or both. While GWAS have not targeted copy number variations (CNVs), a recent study of linkage-disequilibrium between common SNVs and common CNVs has concluded that “..for complex traits, the heritability void left by genome-wide association studies will not be accounted for by common CNVs.” [14]. Our analysis is insensitive to the nature of the inherited variations but we can conclude that they must be rare to have escaped detection in large scale studies, and must have large effects to account for the separation between modes of *Adiposity* (0.93 SD). It may be that a large number of dominantly-expressed risk variants with large effects will be easier to work with than the polygenic alternative, especially if the effects are clustered within a limited number of pathways [48], such as those involved in the regulation of feeding behavior [9].

### Phenotypes

The *Adiposity* factor showed a clearer pattern of responses to the explanatory variables in this analysis than did the alternative measures of body fatness. Although *Adiposity* correlated strongly and equally well with Body Fat % and log-transformed BMI it can be expected to provide more clarity for two reasons. First, factor extraction eliminates uncorrelated components of the input variables, which can result in a reduction in error variance and hence in more statistical power. Second, no assumptions were made about the structure of the factor, except for the log-transformation of the inputs to accommodate the dimensional relationships between them [21], and *Adiposity* may therefore reflect more accurately the biology of fat stores. The clear loading pattern of y-variables on the two factors which leads naturally to their interpretation as *Frame* and *Adiposity*, and the isolation of segregation behavior to *Adiposity* give support to the biological validity of the factors.

In a comparable study Tayo et al [46] analysed a collection of 7 raw obesity-related phenotypes including BMI, BSA, fat mass and %fat, extracting two factors, both of which showed evidence of segregation in families with dominant or additive expression. As the structures of the factors could not be interpreted biologically, the authors concluded that they represent different genetic variants with pleiotropic effects on obesity-related traits. More likely however their factor structures are a product of the inappropriate choice of phenotypes for the analysis, resulting in a correlation structure which violates key requirements for the extraction of reliable and interpretable factors. For example BMI and BSA are both calculated from height and weight, and fat mass and fat% both contain the same primary measurement; inclusion of both members of each pair could cause severe multi-collinearity, as found in our data if analysed in that way (not shown). In addition all of Tayo et al 's obesity-related variables are either surrogates or arbitrary constructs unlikely to relate directly to underlying biology, and fat mass contains a component related to body size for which there is no clear marker amongst the variables. We can reproduce the results of Tayo et al [46] as they relate to dominant or additive expression of segregating obesity susceptibilities but, through a more rigorous approach to phenotype construction, we provide a simpler, biologically plausible explanation. *Adiposity* appears to capture more accurately the biology of fat stores and to be more genetically informative than the other traits analysed here. It is likely that in genetic studies the increased cost of more accurate phenotyping compared to cheaper surrogate measures would be more than compensated for by the decreased sample size required and by the increased clarity of the results.

### Relationship to overweight/obesity

The estimated proportion of FH− with the high *Adiposity* phenotype (16%), and by implication carrying the risk alleles, is equivalent to the proportion of obese (BMI≥30) individuals in the group (15%). While we did not find explicit statistical support for the presence of multiple modes within our FH− data, previous studies of BMI in much larger samples have detected multiple modes (2 or 3) in different populations, with proportions in the higher modes well within our confidence interval [49], [50]; both studies supported an interpretation of environmentally determined expression of genetic susceptibilities due to major gene effects at the population level. Our result in FH− is also consistent with this interpretation, and together with the segregation of the high *Adiposity* phenotype in FH+ suggests that rare dominantly-expressed risk variants may account for a large fraction of the highly prevalent obesity in non-diabetic as well as diabetic humans.

### Relationship to T2DM

The predicted 85% of unobserved T2DM individuals carrying these variants is close to the estimated 80–90% prevalence of overweight/obesity in T2DM [2]. We conclude that most if not all of the overweight/obesity in T2DM is due to a dominantly expressed genetic susceptibility to increased adiposity. The understanding that subjects who will develop T2DM carry a strong genetic predisposition to increased weight gain as part of the inheritance [8] could do much to alleviate the blame and guilt currently associated with the weight gain in such people and sharpen the focus on early preventative and therapeutic interventions. The pathological impact of such obesity is underlined by its strong association with T2DM and its major metabolic complications.

The relationship between the *Adiposity* variants and the T2DM susceptibility variants also present in FH+ is unclear, in part because the mode of inheritance of T2DM susceptibility is unclear. The results of GWAS imply that with few exceptions (eg FTO [51]) T2DM susceptibility is inherited independently of obesity susceptibility and under those conditions, and assuming a heterogenetic model of T2DM genetics, approximately 30% (0.59×0.5) of our population of FH+ would be at high risk of developing T2DM in their current environment. However as the currently identified SNPs from GWAs represent only a small fraction of the total heritability of both T2DM and obesity they may be unrepresentative and some unknown fraction of shared inheritance (ie pleiotropy) may be involved. In support, a linkage study, which unlike GWAS could detect rare susceptibility variants, found suggestive evidence of pleiotropic inheritance in 6 of 12 loci identified [52]. With full pleiotropy approximately 60% (59%) of the FH+ would be at high risk of developing T2DM. We therefore estimate that FH+ confers between 30% and 60% risk of developing T2DM in this population and environment. Concordance rates of T2DM in DZ twins, who share the same fraction of genes as do 1^st^ degree relatives, range from 3% to 71% [32], [53]–[57], but interpretation is complicated by marked variation in age at disease onset and study ascertainment of disease, and by possible effects of twin status on T2DM risk [57].

### Clinical and public health implications

In a society where the current belief is that voluntary “overeating” leads to weight gain in the majority of people who develop T2DM, the understanding that excess weight is a genetic disorder linked with the T2DM would lift a burden of guilt from the patient and allow earlier targeted therapy. If, as we suggest, similar mechanisms are responsible for a large part of endemic obesity this ought to inform clinical and public health interventions.

### Assumptions & limitations

This is a moderate sized and probably ethnically diverse sample recruited primarily to detect any underlying abnormalities in healthy T2DM relatives and therefore it may not adequately represent the population from which it was drawn, or other populations. Obese subjects (BMI≥30 kg/m^2^) are under-represented in the FH− group (15%) compared to the population of the state of New South Wales (28% [58]), which could reflect the characteristics of the sub-population sampled from and/or the selection criteria used in the studies which provided our data. The moderate sample size is reflected in the wide confidence intervals around some estimates. Replication of the findings in larger samples and with appropriate sampling strategies is indicated.

The interpretation of family history as a purely genetic effect depends on the assumption that persistent effects of shared family environment are negligible in this context. If this assumption is false it would lead to an overestimation of genetic effects in the data. However many well-powered studies have found negligible (if any) effects of shared environment on traits related to body composition or T2DM (see Methods). While we cannot exclude a small contamination of genetic effects with those due to shared environment, published direct evidence demonstrates a predominantly genetic influence of family history in this context.

We also assume that obesogenic environmental influences are saturating in our and similar populations, exposing genetic susceptibilities and permitting an analysis which ignores gene-environment interactions. Violation of this assumption would lead to an underestimation of genetic susceptibility. The apparent plateauing of BMI trends in children in developed countries has two plausible, non-exclusive explanations [27]: the intervention hypothesis, which assigns some of the trend to public health campaigns and interventions, and the saturation equilibrium hypothesis which we assume. While some component due to intervention cannot be excluded, it is unlikely to be a major contributor given the minimal effects so far of recent public health interventions directed at obesity [59]. Moreover, the increase in genetic variance across levels of adiposity seen in a large sample of twins is consistent with greater expression of obesity-susceptibility genes in more obesogenic environments [29].

Within the genetic model of FH, accepting the segregation of *Adiposity* and its implications for susceptibility variant frequencies, we find little missing genetic effect. Hence we conclude that our analysis supports the saturation equilibrium hypothesis as an explanation for plateauing BMI trends in this and other developed countries [27].

The calculation of risk allele and genotype frequencies in the unobserved T2DM-affected family members is based on three additional assumptions: 1) that the FH− group represents the spouse population, 2) that there is random mating in relation to obesity susceptibility between T2DM and spouses and 3) that relative risk ratios reflect accurately the relative proportion of individuals under the higher *Adiposity* mode in FH+ and FH−. The calculations are not in fact very sensitive to plausible violations of the 1^st^ assumption; e.g. if we replace our estimate of α _FH−_ (0.16) with the proportion of obese subjects in the New South Wales state population (0.28) assuming a proportionate change in allele frequency, the estimate of α_T2DM_ is only marginally reduced (from 0.86 to 0.80). The 2^nd^ assumption is supported by very low published estimates (∼2% of variance) of the contribution of assortative mating to transmission of obesity susceptibility [43], [60]. The 3^rd^ assumption implies that *Adiposity* in FH- has the same distributional characteristics as in FH+, which is supported by the large-scale studies previously cited [49], [50].

## Conclusion

Our analysis supports the hypothesis that genetic susceptibility to increased adiposity in T2DM, and perhaps more generally, is predominantly the result of a large number of rare genetic variants with large effects. Although our conclusions are dependent on assumptions, the most important assumptions are well supported by the literature. Clearly these findings must next be replicated in different groups, but meanwhile they strongly suggest a need for a change in approach to gene discovery in T2DM and obesity, to one which places more emphasis on accurate phenotyping and family studies, aims to pool the effects of closely-related rare variants in analyses, and which places more weight on the biological plausibility of proposed links to disease processes [61]. The potential reward is that results may then offer a rational basis for targeting therapy in a manner not yet possible.
